# Precisely Automatic Time Window Locating for an Interferometric Fiber-Optic Sensor Array Based on a TDM Scheme

**DOI:** 10.3390/s18103548

**Published:** 2018-10-19

**Authors:** Ke Cui, Zhongjie Ren, Jieyu Qian, Wenjun Peng, Rihong Zhu

**Affiliations:** 1 The MIIT Key Laboratory of Advanced Solid Laser, Nanjing University of Science and Technology, Nanjing 210094, Jiangsu, China; 13104002376@njust.edu.cn (Z.R.); qianjieyu12@163.com (J.Q.); 18795960774@163.com (W.P.); zhurihong@njust.edu.cn (R.Z.); 2 The Advanced Launching Co-Innovation Center, Nanjing University of Science and Technology, Nanjing 210094, Jiangsu, China

**Keywords:** fiber-optic sensor, time division multiplexing (TDM), cross correlation, time window, interrogation controller

## Abstract

Interferometric fiber-optic sensors are often organized in the form of large-scale arrays by lending the technique of time division multiplexing (TDM) to reduce the system cost. Discriminating the time windows for different sensor units is the prerequisite to successfully demodulate the sensing message, but it traditionally calls for a very time-consuming manual calibration process. To combat this problem, a novel automatic time window locating method is proposed in this paper. It introduces the concept of shape function and carries out the cross-correlation operation between the shape function and the sensor signal. The shape function is defined as the function whose curve profile reflects the main data characteristics of the sensor signal. The time window information is then extracted from the correlation result. This whole process is carried out automatically by the interrogation controller of the sensor system without any manual intervene. Experiments are conducted to validate this method. The proposed method can greatly reduce the complexity of locating time windows in large-scale TDM sensor arrays, and make the practical use of the TDM scheme much more convenient.

## 1. Introduction

Interferometric optical fiber sensors are usually [1,2] organized in the form of large-scale arrays to help reduce cost. Several most famous techniques to realize the sensor array include the wavelength division multiplexing (WDM), the time division multiplexing (TDM), the frequency division multiplexing (FDM), the spatial division multiplexing (SDM) and the coherence-domain multiplexing (CDM) schemes. Among them, the TDM scheme demands very little extra building components, and becomes one of the most efficient and welcoming techniques [3,4]. By utilizing the TDM scheme, one has to resolve the valid time window for each of the sensors in the array, and only by doing that can the sensing measurand carried by the sensors be further retrieved from their corresponding windows.

Generally, there are three widely used signal demodulation methods to faithfully calculate the sensing measurand, called the heterodyne [5,6], the phase generated carrier (PGC) [7] and the 3×3 coupler [8,9]. In addition, a new demodulation method called the “rectangular-pulse binary (RPB) phase modulation” was recently proposed [10] by our group. Based on this modulation scheme, a highly compact and efficient interrogation controller based on a field programmable gate array (FPGA) was developed [11]. Although the RPB scheme owns the benefits of high efficiency and low implementation complexity, the original design involves a very time-consuming manual calibration process aiming to find the time windows of the sensors, which somehow offsets its above benefits. Specifically, the calibration process needs to abort the normal working mode and upload massive additional data which contain the whole information of the sensor signal and the synchronization signal. Then, the relative delays between the sensor time windows and the synchronization signal are determined by manually evaluating their distances along the time axis. In order to facilitate the calibration process, by observing the data characteristics of the sensor signal, a novel automatic time window locating method based on a cross-correlation principle is proposed and experimentally validated in this paper. The novelty of the method is the introducing of a shape function whose curve profile accords with the main characteristics of the sensor signal. This shape function is initially phase aligned with the synchronization signal, and then right shifted gradually along the time axis aiming to overlap with the sensor signal. When the two curves overlap, the obtained shifting value equals the delay information that needs to be calibrated. The right shifting and curve comparing tasks are proposed to be fulfilled by conducting cross-correlation between the shape function and the sensor signal and then searching for the maximal value. Utilizing this novel method, the delay information can be automatically calibrated without any manual intervention, making the practical use of the optical fiber sensor system much more convenient.

## 2. Principle of the RPB Scheme and the Sensor Time Windows

**Figure 1:**
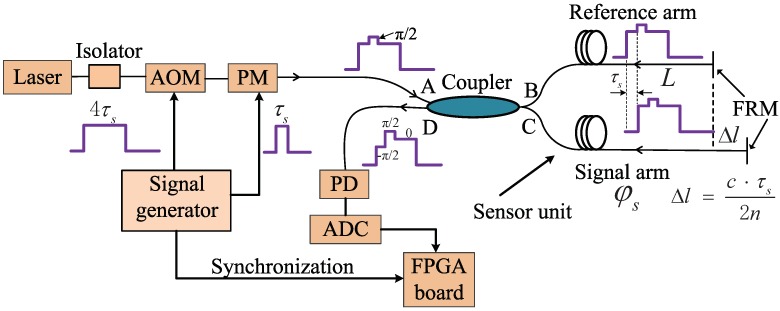
The principle of the RPB scheme.

The working principle of the RPB scheme is depicted in Figure 1. To support the TDM scheme, the continuous light from the laser is firstly chopped into pulses by an acousto-optic modulator (AOM). Then, the light pulses pass through a phase modulator (PM) to obtain an encoded phase shift of $\frac{\pi }{2}$. Both the AOM and the PM are driven by a signal generator. If the duration of the PM signal is set as $\tau_{s}$, the duration of the AOM signal should be at least $4\tau_{s}$ so that the final interferometric signal can contain three different encoded phase shifts of $-\frac{\pi }{2}$, $\frac{\pi }{2}$ and 0 (see Figure 1). The AOM signal can be viewed as consisting of four time slots, each of which is $\tau_{s}$ long. The PM signal should be controlled to be applied to the second time slot of the AOM signal. The sensor unit in Figure 1 is actually a Michelson interferometer [12] with unbalanced arm lengths, whose path difference is $\Delta l=\frac{c\cdot \tau_{s}}{2n}$, where *c* is the light speed in vacuum and *n* is the effective refractive index of the fiber. The split light after the coupler propagating along the longer optical arm (acting as the signal arm) experiences a longer delay time ($\tau_{s}$) relative to the split light along the shorter optical arm (acting as the reference arm). When combining at the D port of the coupler, the light beam from the signal arm carries an encoded phase shift sequence of $\left(0,\;\frac{\pi }{2},\;0\right)$, while the light beam from the reference arm carries an encoded phase shift sequence of $\left(\frac{\pi }{2},\;0,\;0\right)$. Thus, the combined light will have an encoded phase shift sequence of ($-\frac{\pi }{2}$, $\frac{\pi }{2}$, 0). In addition, a synchronization signal which is generated synchronously with the driven signals to the AOM and PM is provided to serve as the time reference for the sensor signal.

The light intensity *I* received at the photo detector (PD) can be expressed as:

(1) $$I=A+AV\cos(\varphi_{s}+\varphi_{e})$$

with

(2) $$\varphi_{e}\left(t\right)=\left\{\begin{array}{cc} -\frac{\pi }{2} & \;\;\;\;\;k\cdot T+\tau_{delay}<t\leq k\cdot T+\tau_{delay}+\tau_{s},\\ \frac{\pi }{2} & \;\;\;\;\;k\cdot T+\tau_{delay}+\tau_{s}<t\leq k\cdot T+\tau_{delay}+2\tau_{s},\\ 0 & \;\;\;\;\;k\cdot T+\tau_{delay}+2\tau_{s}<t\leq k\cdot T+\tau_{delay}+3\tau_{s}, \end{array}\right.$$

where *A* denotes the light power, *V* denotes the fringe visibility, $\varphi_{s}$ is the phase signal that reflects the sensing measurand, $\varphi_{e}$ is the encoded phase shift, *k* is the number of the interrogation pulse, *T* is the period of interrogation pulse, and $\tau_{delay}$ is the delay between the signal time window and the synchronization signal. The existence of $\tau_{delay}$ necessitates the implementation of the calibration process to guarantee the correct time window locating. The *I* signal is sampled by an analog-to-digital converter (ADC) and then transferred to an FPGA board for signal processing. The FPGA also receives the synchronization signal from the signal generator to label the starting moment of each interrogation period. The FPGA should split three intensity data flows from *I* according to the values of $\varphi_{e}$ making that $I_{1}=A+AV\cos\left(\varphi_{s}-\frac{\pi }{2}\right)$, $I_{2}=A+AV\cos\left(\varphi_{s}+\frac{\pi }{2}\right)$ and $I_{3}=A+AV\cos\varphi_{s}$. By conducting, it holds that:

(3) $$\varphi_{s}^{W}=\arctan\frac{I_{1}-I_{2}}{2I_{3}-\left(I_{1}+I_{2}\right)},$$

where $\varphi_{s}^{W}$ is the principle value of $\varphi_{s}$ in the range of $\left(-\frac{\pi }{2},\;\frac{\pi }{2}\right)$. To recover $\varphi_{s}$ from $\varphi_{s}^{W}$, some phase unwrapping algorithm can be applied. Generally, the phase unwrapping algorithm can be classified into three categories: (1) path following method [13]; (2) minimum norm method [14]; and (3) Bayesian algorithms [15]. The applied phase unwrapping algorithm in this paper is the same as in [11] that belongs to the path following method category.

For the TDM-based sensor array with *N* sensor units, there are 3N data flows in total that should be split by the FPGA, and the light intensity $I_{j,i}$ can be generally expressed as:

(4) $$I_{j,i}=A_{j}+A_{j}V_{j}\cos\left(\varphi_{s,j}+\varphi_{e,i}\right)\;\;\;\left(j=1,2\ldots ,N;\;\;\;i=1,2,3\right),$$

where $A_{j}$ denotes the light power for the *j*-th sensor unit, $V_{j}$ denotes the fringe visibility for the *j*-th sensor unit, $\varphi_{s,j}$ is the phase signal for the *j*-th sensor unit, with $\varphi_{e,1}=-\frac{\pi }{2}$, $\varphi_{e,2}=\frac{\pi }{2}$, and $\varphi_{e,3}=0$.

**Figure 2:**
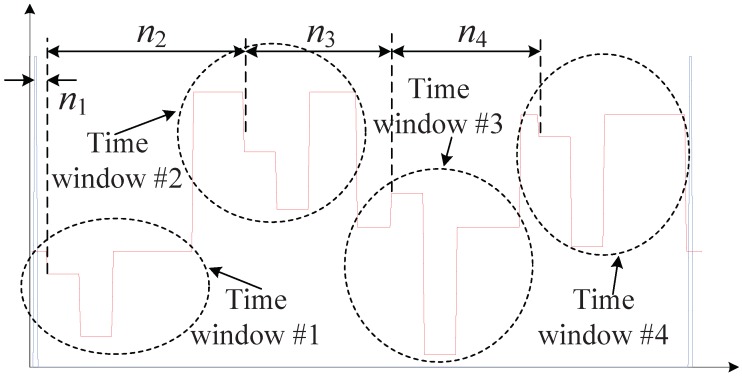
Time windows for a practical TDM sensor array.

The example curve of the *I* signal (in red) and the synchronization signal (in blue) for a four-sensor array is depicted in Figure 2. As denoted in the figure, the parameter $n_{j}$ (j=1,2,3,4) needs to be determined to extract all the intensity data flows. Here, $n_{1}$ represents the time delay between the first senor unit to the synchronization signal, and $n_{j}$ (j=2,3,4) represents the time delay between the *j*-th sensor unit to the (j−1)-th sensor unit. The $n_{j}$ (j=2,3,4) is a known constant value once the sensor array is set up, since it is definitely determined by the fiber distance between adjacent sensor units. However, $n_{1}$ is completely unclear, since the *I* and synchronization signals experience several different optical and electrical components before reaching the FPGA board, causing the time delay not easy to be traced. Thus, the task to locate the time windows for the sensor array is reduced to calibrate the value of $n_{1}$. After obtaining $n_{1}$, the time windows for the *j*-th sensor can be determined as: $\sum_{i=1}^{j}n_{i}+\frac{\tau_{s}}{2}$, $\sum_{i=1}^{j}n_{i}+\frac{3\tau_{s}}{2}$ and $\sum_{i=1}^{j}n_{i}+\frac{5\tau_{s}}{2}$. With all this delay information of the time windows, all of the light intensity $I_{j,i}$ (j=1,2…,N;i=1,2,3) can be extracted and then phase demodulated. As mentioned above, the traditional calibration process needs to abort the normal working mode, upload massive additional data to construct the similar curve as in Figure 2 to visualize the timing relationship, and finally manually evaluate $n_{1}$.

## 3. The Cross-Correlation-Based Automatic Time Window Locating Method

**Figure 3:**
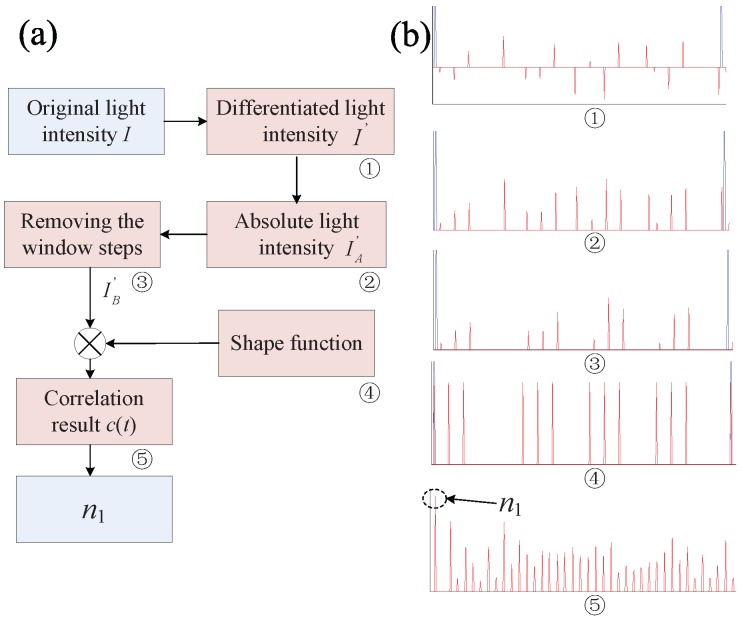
The automatic time window locating method. (**a**) the implementation procedure; (**b**) the temporal implementation result example.

Our goal here is to develop an automatic calculation algorithm to replace the manual intervention process to acquire $n_{1}$. A novel automatic time window locating method based on the cross-correlation principle is proposed, whose implementation procedure is depicted in Figure 3a. The specific operations are described as follows:

1. The original light intensity *I* is firstly self-differentiated by applying $I^{{}^{\prime }}\left(t\right)=I\left(t\right)-I\left(t+\Delta t\right)$, where Δt is the sampling period of the used ADC. The ➀ subfigure in Figure 3b is obtained from Figure 2. By doing this, the influence of $A_{j}$ is eliminated, and the main data characteristics that are reflected by the emerging data spikes are emphasized. These data spikes can be divided into two groups: one reflecting the data jump caused by the three encoded phase shifts (called the signal spike), and the other reflecting the data jump caused by the different $A_{j}$ values for different sensor time windows (called the window spike). Considering that, in the practical sensor system, various types of noises may exist, so a proper threshold value $I_{thd}$ is chosen, and only the data jumps larger than $I_{thd}$ are conceived as valid data spikes.
  How to choose the proper $I_{thd}$ is dependent on the specific noise performance of the given sensor system. The overall noise may be contributed by the laser source, the optical components (AOM and PM), the effect of the temperature and (or) polarization state change, the electrical circuits (PD and ADC), etc. For the sensor system that applies the TDM scheme, the noise performance deteriorates even more due to the noise aliasing effect [16]. To fully investigate the noise performance of the sensor system is not an easy work and beyond the scope of this paper. References [16,17] give a comprehensive analysis to all of the potential noise sources and their contributions to the overall noise performance. Since most of the noise sources accord with the Gaussian characteristic, we heuristically propose to set $I_{thd}=2I_{noi}+3\sqrt{2}\sigma_{noi}$, where $I_{noi}$ represents the measured average noise intensity and $\sigma_{noi}$ represents the measured standard deviation of the noise intensity. This can filter out more than 99% fake spikes generated by the noise signal from the valid data spikes. Since the noise signal is independent from time and, according to the random variable theory, the differentiation operation in the method should add the coefficient 2 to the average variable $I_{thd}$ and $\sqrt{2}$ to the deviation variable $\sigma_{noi}$. Additionally, to assure that no signal spikes are eliminated so that the following operations can be correctly performed, the following criterion should be satisfied:
  (5) $$I_{thd}<I_{spk,min},$$
  where $I_{spk,min}$ denotes the minimum value of the signal spikes. Equation (5) can be guaranteed by choosing low noise component to construct the sensor system (to decrease $I_{thd}$) and (or) inserting optical amplifier to enlarge the light power (to increase $I_{spk,min}$).
2. The absolute value of $I^{{}^{\prime }}\left(t\right)$ is calculated: $I_{A}^{{}^{\prime }}\left(t\right)=\left|I^{{}^{\prime }}\left(t\right)\right|$. The ➁ subfigure in Figure 3b shows the corresponding result.
3. The window spikes are removed from $I_{A}^{{}^{\prime }}\left(t\right)$ to obtain $I_{B}^{{}^{\prime }}\left(t\right)$ by replacing them with zero value. The window spikes can be easily discriminated from the signal spikes, since each signal spike group contains three spikes with an adjacent spike interval equal to $\tau_{s}$, but the window spike group contains only one single spike and is separate from the signal spikes. The ➂ subfigure in Figure 3b shows the corresponding result.
4. A shape function g(t) resembling the signal data curve profile (the signal spikes in the RPB scheme) is needed. The function can be described by letting its functional value be one at the spike positions while zero at other positions. It is mathematically defined in the following form:
  (6) $$\begin{array}{c} f\left(t\right)=\sum_{j=1}^{N}\left(\delta \left(t-n_{j}^{{}^{\prime }}\right)+\delta \left(t-n_{j}^{{}^{\prime }}-\tau_{s}\right)+\delta \left(t-n_{j}^{{}^{\prime }}-2\tau_{s}\right)\right),\;\;\;\\ \mathrm{with}\;n_{1}^{{}^{\prime }}=0\;\mathrm{and}\;n_{j}^{{}^{\prime }}=n_{j}\;\left(j=2,\ldots ,N\right), \end{array}$$
  (7) $$\begin{array}{c} g\left(t\right)=\sum_{l=1}^{p}f\left(t-l\cdot T\right), \end{array}$$
  where δ(t) is the Dirac Delta function, and *p* denotes the total period number. The $n_{1}^{{}^{\prime }}$ is set to 0 to be phase aligned with the synchronization signal. The g(t) is defined as holding a very similar function shape as the ➂ subfigure. The ➃ subfigure shows the g(t) given N=4 and p=1 (corresponding to the example curve in Figure 2).
5. Correlation operation is applied between g(t) and $I_{B}^{{}^{\prime }}\left(t\right)$ to generate the cross-correlation function c(t), which is:
  (8) $$c\left(t\right)=\int_{-\infty }^{+\infty }I_{B}^{{}^{\prime }}\left(\tau +t\right)g\left(\tau \right)d\tau .$$
  By shifting g(t) along the time axis and comparing it with $I_{B}^{{}^{\prime }}\left(t\right)$, the $n_{1}$ is acquired when the two curves overlap. This process can be mathematically accomplished by conducting the cross-correlation operation between the two curves. The $n_{1}$ is the time tick that gives the maximal value of the cross-correlation result. The ➄ subfigure in Figure 3b shows the corresponding correlation result. This step is the most computation-exhaustive part of the proposed method. Equation (8) holds the computational complexity of $O(T^{2})$ for the sensor signal with *T* data points in total. The *T* is proportional to the period number *p*. The *p* should be set to at least 2 to faithfully calculate Equation (8). Larger *p* can give more reliable results but result in a longer calculation time. The *p* can be chosen to be several ones to several tens in practical use.
6. Find the time tick $t_{max}$ that generates the maximal value of c(t), and then $n_{1}=t_{max}$.

Although the proposed automatic time window locating method is for the RPB scheme, the core idea behind the method can be extended to other modulation schemes. As long as apparent data characteristics (such as the signal spike group in the RPB scheme) can be exploited, a shape function resembling the data profile can be constructed, and, by applying cross-correlation, the $n_{1}$ can be automatically determined through finding the time tick that generates the maximal correlation result.

## 4. Implementation and Results

**Figure 4:**
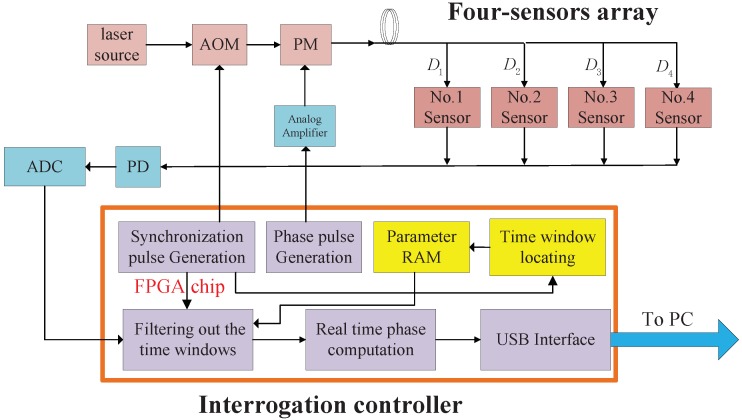
The experimental configuration of a four-sensor TDM array.

The experimental configuration is depicted in Figure 4 to validate the proposed automatic time window locating method. The laser source emits continuous light with 1551.7 nm central wavelength and less than 10 KHz linewidth. The used AOM is driven by the synchronization pulse signal with 400 ns duration and 500 KHz repetition rate. The PM is driven by the phase pulse signal with 100 ns duration and also 500 KHz repetition rate. Each of the four sensor units in the sensor array is fabricated as described in [12] to measure the acceleration and has an approximate sensitivity of 3400 rad/*g*, where *g* represents the gravity acceleration. The sensors are placed naturally on ground, and the environmental vibration on ground imposed to the sensors is adequate to generate the data pattern (the signal spikes), which is essential in the proposed time window locating method. Both the driven signals for the AOM and PM and the synchronization signal for “filtering out the time window” module are generated by the FPGA [11] synchronously. The automatic time window locating module (in the yellow rectangle) is added to the FPGA to implement the proposed method, and its temporal operating results are dumped out to the PC by the logic analyzer tool (SignalTap II, Quartus II software Version 13.1, Intel Corp., Santa Clara, CA, USA) to validate the correctness.

**Figure 5:**
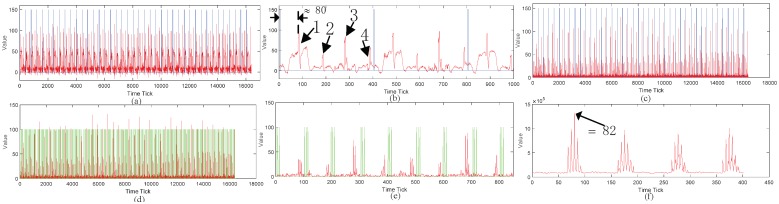
Implementation of the cross-correlation-based automatic locating method. (**a**) the original waveform; (**b**) the zoomed in first two periods of (**a**); (**c**) the calculated $I_{B}^{{}^{\prime }}\left(t\right)$ waveform; (**d**) the $I_{B}^{{}^{\prime }}\left(t\right)$ curve combining the auxiliary shape function wave; (**e**) the zoomed in first two periods of (**d**); (**f**) the calculated cross-correlation results.

Figure 5 shows the gathered implementation curves from the FPGA. Figure 5a shows the actual waveform of I(t). The blue curve represents the synchronization signal and the red curve represents the signal curve. The number of the sensors (or time windows) is four. Figure 5b shows the zoomed in first two periods of Figure 5a. The approximated four time windows are labeled 1, 2, 3 and 4 in the figure and the approximated $n_{1}$ is about 80 by manual inference. Figure 5c is the calculated $I_{B}^{{}^{\prime }}\left(t\right)$ waveform of Figure 5a. Figure 5d substitutes the synchronization signal by the shape function in Figure 5c. Its curve shape can be seen in Figure 5e which contains the zoomed in first two periods of Figure 5d. Figure 5f shows the calculated cross-correlation results between the two curves in Figure 5d. The time tick, which gives the maximal correlation result, is 82 as denoted in the figure. This means that $n_{1}=82$.

**Figure 6:**
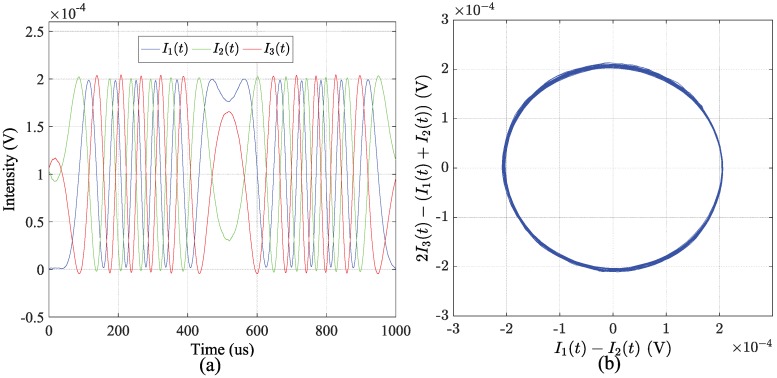
The implementation result of the automatic time window locating method. (**a**) the extracted three intensities; (**b**) the Lissajous curve of $2I_{3}-\left(I_{1}+I_{2}\right)$ versus $I_{1}-I_{2}$.

Then, the three light intensities are extracted out by setting $n_{1}=82$, and they are depicted in Figure 6a. Figure 6b shows the corresponding Lissajous curve of $2I_{3}-\left(I_{1}+I_{2}\right)$ versus $I_{1}-I_{2}$. The Lissajous curve can visually reflect the amplitude ratio, the frequency ratio and the relative phase between the two variables along the *x*- and *y*-axes [18]. The exact circle shape in Figure 6b validates the $\frac{\pi }{2}$ phase shift employed in the RPB scheme and demonstrates that the three light intensities have been correctly extracted out by the automatic time window locating module.

To validate the robustness of the proposed method, the entire sensor array is kept to continuously run for two days under the condition of turning on the integrated automatic time window locating module. The calibrated $n_{1}$ is uploaded to the PC to be recorded every one minute. The sensor signal suffers from different environmental noise changes such as temperature and fiber vibration. All of the obtained $n_{1}$ turned out to be identical and no calibration failure happened. It verifies that the proposed method has very strong noise resistance.

Finally, we make some discussions about the scalability of the proposed method. On one hand, as the sensor number *N* increases, the spikes contained by the shape function (Equation (6)) increase correspondingly. This is beneficial to generate more remarkable peaks in the cross correlation result and make the locating of the time tick $n_{1}$ easier. Thus, from this point of view, this method is friendly to be implemented in large-scale fiber-optic sensor array. However, on the other hand, considering various kinds of noises in the practical sensor system, as the *N* scales up, the noise becomes more significant due to the noise aliasing effect. According to the analysis in [16], the overall noise deviation $\sigma_{noi,N}$ can be approximately evaluated as:

(9) $$\sigma_{noi,N}=4\tau_{s}\cdot N\cdot B\cdot \sigma_{noi,1},$$

where *B* denotes the electrical detection bandwidth and $\sigma_{noi,1}$ denotes the noise deviation of a single sensor system. Equation (9) indicates that the noise deviation $\sigma_{noi,N}$ increases proportionally with *N* and makes Equation (5) tighter to be satisfied. It can be concluded that the proposed method is more beneficial to be applied to the sensor array with a larger scale as long as Equation (5) can be satisfied. Choosing low noise components to construct the sensor system (to decrease $I_{thd}$) and (or) inserting an optical amplifier to enlarge the light power (to increase $I_{spk,min}$) are helpful to survive Equation (5) and the proposed method.

## 5. Conclusions

Locating the time windows for a fiber optical sensor system which is based on TDM is an important step to retrieve the sensing information. However, it lacks an efficient (or convenient) method to fulfill this task up to now. To facilitate the time window calibration process, by taking the RPB modulation scheme as the analysis prototype, we propose a novel automatic locating method. The method introduces the concept of the shape function whose curve profile resembles that of the actual sensor signal (the signal spikes here). Then, by finding the maximal value of the cross-correlation result of the shape function and the sensor signal, the time window information can be determined. An accompanied technique to suppress the influence of noise is also proposed to guarantee the reliability of the method. Long-term experimental results obtained from a four-sensor system validate the effectiveness and robustness of the method. The core idea behind the method can be extended to other modulation schemes. As long as apparent data characteristics (such as the signal spike group in the RPB scheme) can be exploited, a shape function resembling the data curve can be constructed, and the similar automatic time locating procedure can be employed. Exploring the data characteristics in other traditional modulation schemes and extending the proposed automatic locating method to them will be the research focus in our future work.
